# Clinical, Radiological, and Biochemical Evaluation in Orthopedics: Lessons from a Special Issue

**DOI:** 10.3390/biomedicines14092031

**Published:** 2026-09-09

**Authors:** Carlo Biz, Elisa Belluzzi, Pietro Ruggieri

**Affiliations:** 1 Orthopedics and Orthopedic Oncology, Department of Surgery, Oncology and Gastroenterology (DiSCOG), University of Padova, Via Giustiniani 3, 35128 Padova, Italy; carlo.biz@unipd.it (C.B.); pietro.ruggieri@unipd.it (P.R.); 2 Centre for Mechanics of Biological Materials, University of Padova, 35131 Padova, Italy; 3 Musculoskeletal Pathology and Oncology Laboratory, Department of Surgery, Oncology and Gastroenterology (DiSCOG), University of Padova, Via Giustiniani 3, 35128 Padova, Italy

Rather than representing isolated advances, the contributions collected in this Special Issue collectively illustrate the transition of orthopedic evaluation from descriptive assessment toward integrated, quantitative, biologically informed and data-driven medicine. Modern orthopedics is no longer defined solely by surgical innovation, but also increasingly by the ability to understand, measure, and predict musculoskeletal disease through multiple, complementary levels of assessment. Clinical examination, imaging, functional outcomes, biological and biochemical markers, regenerative strategies, and artificial intelligence-based tools are progressively converging toward a more integrated model of patient evaluation [1,2,3,4]. In this context, the central challenge is not only to introduce new technologies into orthopedic practice, but also to determine how they can improve diagnosis, guide treatment planning, assess therapeutic efficacy, and ultimately support more personalized patient care.

Despite remarkable technological advances, orthopedic decision-making still largely depends on integrating heterogeneous sources of information. This Special Issue was conceived to explore how emerging clinical, imaging, biological and computational tools may collectively improve diagnostic accuracy and treatment personalization. The final collection includes seven contributions—four original research articles, two reviews, and one case report—reflecting the breadth and multidisciplinary nature of contemporary orthopedic research. Taken together, these articles address different but complementary dimensions of orthopedic evaluation, ranging from personalized conservative treatment and trauma-related diagnostic challenges to radiological quantification, regenerative procedures, artificial intelligence-based risk prediction, and preclinical assessment of pain and function.

The contribution by Simon et al. [5] addresses the clinical evaluation of conservative orthopedic treatment in patients with patellofemoral pain associated with foot deformity. Patellofemoral pain is typically characterized by anterior, retropatellar, or peripatellar knee pain exacerbated by activities that load the patellofemoral joint, such as squatting, stair ambulation, running, or prolonged sitting [6]. The relationship between altered foot posture and patellofemoral symptoms remains debated, although excessive pronation and foot–ankle alignment have been investigated as potential contributors to lower-limb biomechanical changes and as possible factors influencing the response to foot orthoses [7]. In their randomized controlled study, the authors compare custom-made biomechanical and sensorimotor foot orthoses. Both approaches are associated with pain reduction over a 3-month intervention period, without clear superiority of one treatment over the other. These findings emphasize that, in multifactorial and symptom-driven conditions such as patellofemoral pain, treatment evaluation should consider not only the device or technique itself, but also patient-specific biomechanics, longitudinal symptom changes, and clinically meaningful functional outcomes.

The case report and literature review by Wojtyś et al. [8] draws attention to the diagnostic challenges posed by rare vascular complications in orthopedic trauma. The authors describe a post-traumatic subclavian artery pseudoaneurysm secondary to clavicular fracture, which is initially difficult to recognize because of nonspecific symptoms and misleading imaging findings. Their literature analysis identifies 19 additional documented cases managed through endovascular intervention, providing a broader clinical context for this uncommon but serious condition, including one case managed at our institution [9]. This contribution emphasizes that persistent pain, swelling, or atypical clinical evolution after fracture should prompt careful reassessment, particularly when vascular structures may be involved. It also reinforces the value of multidisciplinary collaboration between orthopedic, radiological, vascular, and trauma specialists in the management of complex injury patterns [10,11].

The need to critically reassess radiological parameters that are routinely used in orthopedic diagnosis is also reflected in the contribution by Schamberger et al. [12]. The authors investigate the radiocapitellar line in healthy elbows and demonstrate that radiocapitellar deviations may be present even in asymptomatic individuals. These findings challenge the use of fixed radiological thresholds alone for diagnosing posterolateral rotatory instability and emphasize the need to combine imaging data with clinical, anatomical, and functional assessments. This contribution is particularly relevant because it reminds clinicians that radiographic parameters, although essential, should not be interpreted in isolation [13].

Calcific tendinopathy of the rotator cuff is commonly evaluated using conventional radiography and ultrasound, which remain central for identifying calcific deposits and assessing their location and morphology. However, the radiological appearance of calcific deposits may vary according to the phase of the disease, and traditional qualitative classifications based on deposit morphology, density, size, or margins have shown limitations in terms of reliability and reproducibility [14]. In this context, Kim et al. [15] explore the radiographic features of rotator cuff calcifications on conventional shoulder X-rays using spatial grayscale analysis. Their work suggests that calcific deposits differ from adjacent tendon tissue not only in brightness but also in spatial heterogeneity, with correlations between radiographic parameters and ultrasonographic findings. This study points toward a more objective and reproducible use of conventional radiography, transforming a widely available diagnostic tool into a source of quantitative information with potential pathophysiological meaning.

Bone marrow aspirate concentrate is not a new concept in orthopedic regenerative medicine, and its use has been explored in several musculoskeletal conditions because of its cellular and biological components [16]. However, important methodological questions remain regarding how bone marrow should be harvested in order to obtain aspirates with favorable cellular characteristics and regenerative potential. Biological and regenerative perspectives are addressed by Che et al. [17], who compare single- and multiple-level bone marrow aspiration techniques from the anterior iliac crest. Their findings suggest that a single-level aspiration method may provide a high-quality bone marrow aspirate with favorable mesenchymal stem cell characteristics and enhanced osteogenic potential compared with the multiple-level approach. This study contributes to the ongoing discussion regarding standardization of bone marrow aspiration techniques and optimization of cell-based strategies in musculoskeletal regeneration.

Another emerging direction is the use of artificial intelligence in orthopedic diagnosis, risk prediction, and clinical decision support. Vulpe et al. [18] review the role of artificial intelligence and machine learning in predicting periprosthetic joint infections, one of the most challenging complications after joint replacement. This topic is particularly relevant because the diagnosis and prevention of periprosthetic joint infection require the interpretation of heterogeneous data, including clinical risk factors, imaging findings, laboratory biomarkers, microbiological results, and perioperative variables. Their review highlights the potential of AI-based models to support early detection, risk stratification, and personalized clinical decision-making, while also acknowledging the current limitations related to data quality, validation, interpretability, and clinical implementation. This contribution reflects the growing relevance of computational medicine in orthopedics and the need for careful translation of digital tools into practice.

Finally, Kloser et al. [19] expand the scope of the Special Issue towards preclinical evaluation by providing a comprehensive review of behavioral, mobility, and pain-related outcomes in rodent models of osteoarthritis induced by destabilization of the medial meniscus. Their analysis shows that, although these models are widely used to study post-traumatic osteoarthritis, the assessment of pain and functional impairment remains highly heterogeneous across studies, with substantial variability in behavioral assays, time points, animal characteristics, and reporting practices. This review is particularly valuable because it shifts attention from structural joint degeneration alone to outcomes that are more closely related to the clinical experience of osteoarthritis, namely pain, mobility limitation, and functional decline. By highlighting the need for standardized protocols and more consistent reporting, the authors provide a useful framework to improve reproducibility and strengthen the translational relevance of preclinical osteoarthritis research.

Overall, the articles collected in this Special Issue show that orthopedic evaluation is becoming increasingly multidimensional. Clinical examination and imaging remain essential, but they are now complemented by quantitative radiological analysis, patient-reported outcome measures, cellular and biochemical characterization, and artificial intelligence-based tools. This integrated approach is particularly important in musculoskeletal medicine, where symptoms, structural damage, biological activity, and functional impairment do not always progress in parallel.

Several common messages can be drawn from the contributions. Diagnostic criteria should be continuously reassessed in light of new evidence, especially when parameters originally considered pathological may also be observed in healthy or asymptomatic subjects. Conventional imaging can gain new value when analyzed quantitatively and correlated with clinical or ultrasonographic findings. Clinical outcomes should remain central to evaluating both conservative and surgical treatments. Regenerative and biological approaches require careful methodological standardization before their full potential can be translated into routine practice. Finally, digital technologies and artificial intelligence offer promising opportunities, but their adoption must be supported by rigorous validation, transparency, and clinical relevance. One of the most striking observations emerging from this Special Issue is that advances in orthopedic diagnosis are occurring simultaneously across traditionally separate disciplines. Clinical medicine, imaging, regenerative biology and artificial intelligence are no longer independent fields but complementary components of a unified diagnostic framework.

In conclusion, this Special Issue provides a multifaceted overview of current advances in clinical, radiological, biochemical, and translational evaluation in orthopedics. The published contributions reflect the complexity of modern musculoskeletal medicine and the need for multidisciplinary collaboration among clinicians, radiologists, biologists, engineers, and data scientists. We hope that this collection will stimulate further research aimed at improving diagnostic accuracy, refining outcome assessment, strengthening collaboration among experts of different backgrounds, and developing more effective strategies for the management of orthopedic patients.

Finally, future orthopedic practice will increasingly rely on the integration of clinical expertise, quantitative imaging, biological characterization, and computational intelligence (Figure 1). The challenge will no longer be the availability of data, but rather their meaningful integration into patient-centered decision making.

## Figures and Tables

**Figure 1:**
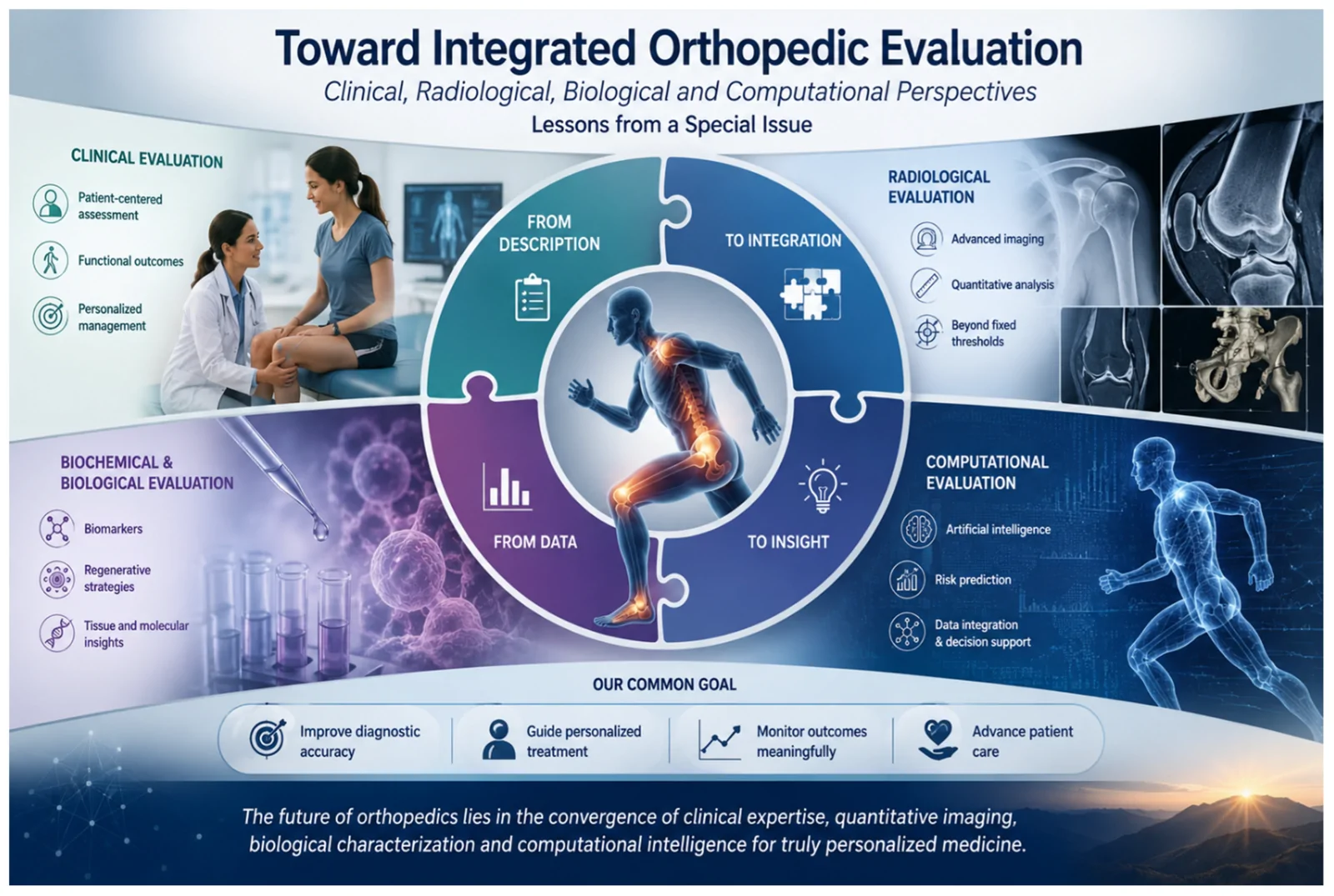
Integrated orthopedic evaluation combining clinical, radiological, biological, and computational approaches to support personalized patient care.
